# Understanding the Dynamics of the Developing Adolescent Brain Through Team Science

**DOI:** 10.3389/fnint.2022.827097

**Published:** 2022-02-22

**Authors:** Kiki Zanolie, Ili Ma, Marieke G. N. Bos, Elisabeth Schreuders, Annelinde R. E. Vandenbroucke, Jorien van Hoorn, Anna C. K. van Duijvenvoorde, Lara Wierenga, Eveline A. Crone, Berna Güroğlu

**Affiliations:** ^1^Department of Developmental and Educational Psychology, Institute of Psychology, Leiden University, Leiden, Netherlands; ^2^Leiden Institute for Brain and Cognition (LIBC), Leiden University, Leiden, Netherlands; ^3^Erasmus School of Social and Behavioral Sciences, Erasmus University Rotterdam, Rotterdam, Netherlands

**Keywords:** adolescence, brain development, social development, mental wellbeing, team science

## Abstract

One of the major goals for research on adolescent development is to identify the optimal conditions for adolescents to grow up in a complex social world and to understand individual differences in these trajectories. Based on influential theoretical and empirical work in this field, achieving this goal requires a detailed understanding of the social context in which neural and behavioral development takes place, along with longitudinal measurements at multiple levels (e.g., genetic, hormonal, neural, behavioral). In this perspectives article, we highlight the promising role of team science in achieving this goal. To illustrate our point, we describe meso (peer relations) and micro (social learning) approaches to understand social development in adolescence as crucial aspects of adolescent mental health. Finally, we provide an overview of how our team has extended our collaborations beyond scientific partners to multiple societal partners for the purpose of informing and including policymakers, education and health professionals, as well as adolescents themselves when conducting and communicating research.

## Introduction

**Figure 1:**
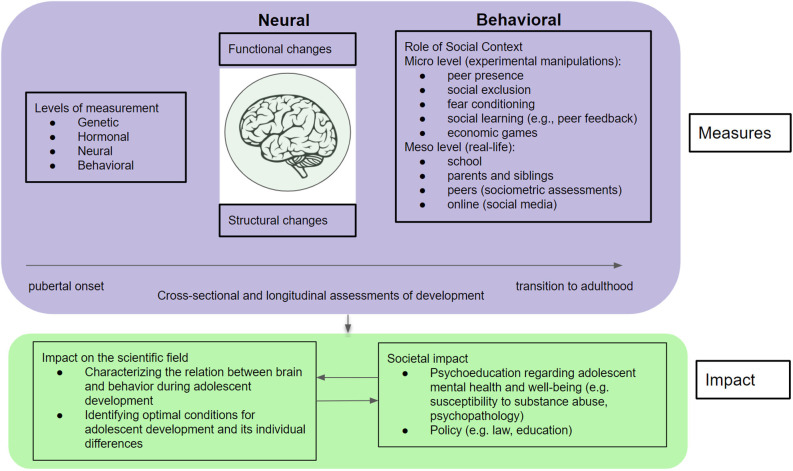
Overview of the measures required to chart the complexity of developmental changes during adolescence and their impact. Note: This figure illustrates the richness of measures needed in studies that aim to capture the complexity of developmental changes during adolescence (purple). Findings from such studies will subsequently have a scientific and societal impact (green). Impact on the scientific field and on society are interlinked, as collaborating and communicating with societal stakeholders (e.g., policymakers, teachers, parents, and adolescents) also informs new research questions.

Adolescence is a developmental phase between the ages of 10 and 24 years (Sawyer et al., 2018). Adolescence starts with puberty, setting off a cascade of hormonal changes signaling the start of biological maturation (Dahl et al., 2018), and is characterized by major physical, psychological, and social changes (Blakemore and Mills, 2014). Adolescents navigate an increasingly complex social network in which peer relations become more salient and are an important source for social learning (e.g., learning about, with, and from peers to adjust to changing social environments; Westhoff et al., 2020a). Both peer relations and social learning have a great impact on mental well-being (Nelson et al., 2005, 2016; Vitaro et al., 2009). Moreover, adolescence is considered a period of heightened sensitivity to mental health problems, with approximately 75% of adult mental health problems first appearing during adolescence (Kessler et al., 2007; Solmi et al., 2021). As biological, psychological, and social changes occur concurrently in adolescence, it is crucial to understand how these changes are intertwined and contribute to successful developmental outcomes, such as resilience and mental health, as well as to maladaptive outcomes, such as risky behaviors and psychopathology (Davey et al., 2008; Crone and Dahl, 2012; Güroğlu, 2021). We further argue that understanding adolescence as a developmental phase with risks and opportunities requires incorporating a transactional perspective with measurements at multiple levels (genetic, hormonal, neural, behavioral) and across different social settings (e.g., school, parent relationships, peer relationships; see Figure 1). Considering the multitude of factors influencing development and the interlinked complexity of their corresponding measurement levels, we propose that team science is a fruitful approach to understanding the dynamics of adolescent development.

To understand individual differences in optimal conditions for growing up in an increasingly complex social world, we use a variety of neurobiological and behavioral methods. Current influential models of adolescent brain development describe an asynchronous development of the limbic “socio-affective system” and cortical “cognitive control system” during adolescence (Steinberg, 2008; Somerville et al., 2010). These models emphasize that faster maturation of the limbic system compared to the slower maturation of the cortical system underlies heightened reward sensitivity and risk-taking tendencies, leading to risky and impulsive behaviors such as alcohol use (Peters et al., 2017). Recent accounts of adolescent development also include the impact of individual differences in hormonal, genetic, behavioral, and neural influences which are intertwined in a social context (Crone and Dahl, 2012; Pfeifer and Allen, 2021). Specifically, adolescence is seen as a time for heightened goal flexibility, where social goals can influence pathways for development. Hence, the asynchronous development between the limbic and cortical system, together with increasingly complex and influential social experiences, such as peer relations and social learning, makes adolescence a sensitive window for socio-affective development which can lead to multiple pathways, such as risky behaviors and mental illness, or prosocial behavior and mental resilience (Crone and Dahl, 2012; Güroğlu, 2021; see Figure 1).

In this article, we highlight the unique position of our highly collaborative multidisciplinary research program and focus on its contributions to the field. We provide an overview of our research focusing on: (1) meso level peer relations; and (2) micro level social learning and next describe their combined influence on mental health. We propose that these two themes are crucial in charting the complexity of the dynamically interlinked biological, psychological, and social changes in adolescence. We provide examples of our research designs with controlled experimental settings at the micro-level and assessments of real-life social relationships at the meso level (see Figure 1). Finally, we demonstrate how collaborations can be extended to multiple societal partners to inform and include policy makers, education and health professionals, and adolescents themselves when conducting and communicating research. We conclude that understanding the complex dynamics of adolescent development requires rich measurements and we highlight the promising role of team science in achieving this goal.

### Peer Relations

Adolescence is characterized by a significant shift in focus from parents toward peers, also referred to as social reorientation (Nelson et al., 2005). Compared to children, adolescents spend increasingly more time without adult supervision and in the company of their peers, where fitting in the peer group and peers’ opinions become vital for adolescents’ self-identity development (Laursen and Veenstra, 2021). Social goals, such as acceptance by the peer group and forming and maintaining friendships, are particularly important in the school context, as they are consistently linked with markers of positive social adjustment and academic achievement (Dawes, 2017). Recently, evidence from neuroimaging studies corroborate the significance of the peer context for adolescents by finding that adolescents show heightened neural responses during social decision-making in brain regions related to reward and motivation, such as the ventral striatum, and in social cognition, such as dorsomedial prefrontal cortex (for reviews see Van Hoorn et al., 2019; Andrews et al., 2021). For example, in early- and mid-adolescence, mere peer presence, when being observed by an unfamiliar peer, results in heightened neural activation in the medial prefrontal cortex (mPFC; Somerville et al., 2013), and when being observed by a friend, adolescents show increased risk-taking behavior, with heightened activation of the ventral striatum (Chein et al., 2011). In an Event-Related Potential (ERP) study, we have shown that manipulation of participants’ social rank (high vs. low rank) modulated neural responses during social exchanges in mid-adolescents but not in children or adults, signifying that even transient social interactions are particularly salient for mid-adolescents (Zanolie and Crone, 2021). In another study, compared to being alone, the presence of a group of spectators (consisting of adolescent confederate actors) led to increased prosocial behavior which was accompanied by enhanced activation in social brain areas such as mPFC, temporal parietal junction (TPJ), precuneus and superior temporal sulcus (STS; Van Hoorn et al., 2016). Taken together, these studies of peer presence illustrate the importance of capturing the peer context when studying adolescents’ social behavior (Figure 1, micro level).

Neuroimaging studies aiming to capture real-life peer context (Figure 1, meso level), however, face the challenge of bringing peer relationships into the highly controlled experimental laboratory setting, such as in the MRI scanner (see Güroğlu and Veenstra, 2021 for a more extensive review of this research line). In tackling this challenge, sociometric assessments of the peer network provide a useful tool to classify an individual’s peer status within a real-life peer group (see Box 1). Combinations of sociometric assessments with neuroimaging and/or economic exchange paradigms assessing social decision-making (see Box 2) led to insights into how social interactions and their neural underpinnings may depend on peer context (Güroğlu et al., 2014). For example, we found in adults that interactions with familiar peers relate to heightened activation of brain regions of affect and reward (including the ventral striatum and amygdala) and social cognition (including the mPFC, TPJ, STS, and precuneus; Güroğlu et al., 2008). Recently, we showed that the developmental trajectories of ventral striatum responses to rewards are modulated by friendship stability across a 5-year period (Schreuders et al., 2018) and that ventral striatum responses to winning money are also (negatively) related to acceptance by the peer group (Meuwese et al., 2018). We also showed that in young adults (Schreuders et al., 2018) and mid-adolescents (Schreuders et al., 2019), prosocial decisions toward friends compared to disliked or unfamiliar peers, are related to increased activation of the putamen, part of the reward circuitry, and the posterior temporoparietal regions that are involved in social-cognitive processes. Moreover, neural responses to social rejection depend on the excluder’s peer status relative to the adolescent’s own status (De Water et al., 2017). Our longitudinal studies further showed that the history of peer experiences across childhood modulates neural responses to social exclusion and during social decision-making in adolescence (Will and Güroğlu, 2016; Will et al., 2016, 2018; Asscheman et al., 2019).

Box 1 **Using sociometric assessments to study social experiences.**

Sociometric assessments based on nominations of classmates on various criteria (e.g., “who do you like?”, “who do you dislike?”, “who are your friends?”) are most valuable for assessing social experiences, and more specifically for assessing peer relationships, in an efficient manner. Crucially, these social assessments require access to a closed network (e.g., classmates, sports team, an orchestra) where the nominations can be made. These nominations can be used to assess social experiences at two levels. At the dyadic level, they reveal reciprocal relationships, such as mutual friendships between two people who nominate each other as a friend (see, e.g., Güroğlu et al., 2008; Schreuders et al., 2019). At the group level, they reveal information on the status of the individual within the peer group, such as accepted or rejected status based on the total number of received nominations from like and dislike nominations (Will et al., 2016; Will and Güroğlu, 2016). Finally, by applying graph theory, these nominations can be used to calculate social network characteristics that can be used to characterize individuals’ positions in the network, such as centrality, as well as identifying group level characteristics, such as social cohesion (Van den Bos et al., 2018). An increasing number of studies combine assessments of peer relations with neuroscientific designs to investigate how peer relations modulate brain activity (see for review Güroğlu and Veenstra, 2021).

Box 2 **Using economic games to study social interactions.**

Economic games create social contexts that reveal fundamental aspects about the participant’s social preferences in ways that are quantifiable. They have proven highly efficient in assessing various forms of (pro) social behavior both in adults and in development (Camerer, 2011; Will and Güroğlu, 2016). These paradigms are based on an economic exchange between at least two players where the participant’s decisions have actual consequences for their own and their interaction partner’s payoff. The simplest example is the *Dictator Game* in which the first player is given valued goods (e.g., money, toys, candies) and can share a portion of those goods with a second player. While game theoretical models assume that humans are rational players who aim to maximize their own profit, findings consistently show that people typically share some portion of the goods, thereby revealing other-regarding preferences. In the *Ultimatum game*, the first player (proposer) is a variant where the second player can either accept or reject the share given by the first player. If accepted, the goods are divided as proposed by the first player. If rejected, both players receive nothing. The reward maximizing strategy is to accept any offer greater than zero, but again consistent findings show that offers viewed as unfair are rejected (Fehr and Schmidt, 1999). Offers made by the first player are typically higher in the Ultimatum Game than Dictator Game, revealing strategic considerations to reduce the probability of rejection. Another variant is the *Trust Game*, in which the first player (investor) can again share a portion of goods with the second player (trustee). The portion received by the trustee is multiplied by the experimenters. The trustee then chooses to either share the profit with the investor (reciprocation; both profit from the exchange) or keep all the profit (betrayal; only the trustee profits, the investor loses the entrusted amount; Berg et al., 1995). These paradigms can be presented in a *repeated* fashion, such that participants play multiple rounds of these games with the same partner(s). Feedback received during these repeated interactions facilitates learning about the social preferences of other individuals or groups. Economic exchange paradigms are simple enough to administer to a wide age range (from 3 years old to adults) and enable studying developmental patterns in social behavior (Güroğlu et al., 2009; Meuwese et al., 2015; Zanolie et al., 2015; Ma et al., 2017). Moreover, their structured nature makes them further suitable for neuroimaging research.

Taken together, increasing evidence shows both current and long-term patterns of social experiences with peers modulate adolescent social behavior and their underlying neural processes (Güroğlu, 2022). In order to understand the developing brain in a social context, future studies need to incorporate measures of social networks with assessments of brain function and structure (Lamblin et al., 2017; Baek et al., 2021). Additionally, the complexity of social dynamics has in recent years only been amplified through the addition of the online social layer where young people can have meaningful connections. Future studies aiming to understand the dynamics of adolescent development need to include assessments of both offline and online connections.

### Social Learning

Peer relations interact with individual and social learning. Social learning encompasses learning about, with, and from others. In the peer context, it involves learning about the characteristics and preferences of a peer or a peer group, such as their trustworthiness or cooperativeness (Nelson et al., 2005; Blakemore and Mills, 2014; Sawyer et al., 2018). Adaptive social behavior requires adolescents to learn about these characteristics and adjust their own behavior accordingly, such as learning when to be prosocial and towards whom (Steinberg and Morris, 2001; Van den Bos et al., 2011; Lockwood et al., 2016; Crone and Fuligni, 2020). These social learning processes are crucial for fostering healthy relationships with peers, which are predictive of adolescents’ long-term well-being (Paus et al., 2008; Crone and Dahl, 2012; Dahl et al., 2018; Sawyer et al., 2018).

Social learning is often studied with repeated behavioral economic paradigms, where participants play multiple rounds of an economic game with the same partner or multiple partners from one experimentally selected group (see Box 2). Peer characteristics or peer evaluations are typically experimentally manipulated, allowing participants to learn through positive and negative feedback (Ma et al., 2020; Westhoff et al., 2020b; Zanolie and Crone, 2021). Reinforcement learning models can then be used to characterize individual differences in learning strategies, learning speed, and the underlying cognitive processes that cause age differences in learning about others (Sutton and Barto, 2018; Nussenbaum and Hartley, 2019; Wilson and Collins, 2019). For example, we used an information sampling paradigm in which participants were able to sample information about a peer’s history of trustworthiness before deciding to trust or not trust them (Ma et al., 2020). We found that behavioral adaptation to the gathered evidence improved with age, especially from early to mid-adolescence. In other studies, we manipulated the cooperativeness of groups (Westhoff et al., 2020b) and used a probabilistic learning task in which participants could sometimes earn rewards for themselves and sometimes for others (Westhoff et al., 2021). We found that probabilistic learning to benefit others showed age-related improvement across adolescence and was associated with ventromedial prefrontal cortex responses to unexpected outcomes. Learning for the self was stable across adolescence and associated with ventral striatal responses to unexpected outcomes. Together, these findings suggest that adolescents show rapid improvements in behavioral adjustments to the social environment, especially from early to mid-adolescence. These findings are consistent with the idea that learning about the consequences of actions in an interpersonal context is especially salient for adolescents and furthermore highlight early to mid-adolescence as a sensitive period for learning about others (Blakemore and Mills, 2014; Nelson et al., 2016; Sawyer et al., 2018; Andrews et al., 2021).

Considering that learning at school takes place in the peer context (i.e., in classrooms and group assignments), learning about, with, and from peers are also crucial research lines to identify the optimal conditions of learning at school. In ongoing studies, we focus on determining which individuals work well together by combining reinforcement learning or feedback processing paradigms with sociometric assessments. Such studies form the first steps of identifying optimal conditions for learning in the context of peers by informing how peer relationships in classrooms and differences in learning strategies between collaborating students may influence (social) learning.

### Social Experiences and Mental Health

It is well-established that social experiences influence mental health and well-being. For example, close friendships during adolescence are a protective factor against mental health problems across adolescence and later in life (Van Harmelen et al., 2017, 2021). However, being rejected by peers is associated with self-harm (Esposito et al., 2019) and depressive symptoms (Platt et al., 2013). Also, epidemiological studies have shown a peak in the emergence of mental health problems across adolescence (Dalsgaard et al., 2020). Showing symptoms of psychopathology in childhood or adolescence are found to be a key predictor of mental health problems and other adverse outcomes later in life (Zisook et al., 2007; Caspi et al., 2020). These findings indicate that there are developmental processes enhancing or bringing about vulnerabilities to develop mental health problems. Theoretical models propose a complex interplay between brain development, hormonal changes, and social development in interaction with environmental factors that may explain the emergence and maintenance of mental health problems across adolescence (Pfeifer and Allen, 2021). So far, only a few studies have directly linked the relationship between social context, brain development, and mental health outcomes. One study showed that greater subgenual anterior cingulate activity (sgACC) during a social exclusion game was associated with an increase in parent-reported depressive symptoms 1 year later (Masten et al., 2013). Social interactions with friends have also been related to activation of the sgACC and the ventral striatum. These brain regions are associated with the reward circuitry, speculatively providing indirect evidence linking positive peer interactions with mental health (Güroğlu et al., 2008; Schreuders et al., 2021). Research is needed to elucidate the complex interplay between brain development, social context, and mental health (Davey et al., 2008; Pfeifer and Allen, 2021). To better understand mental health and the transition from mental health to mental illness, our ongoing studies aim to contextualize individual differences in relation to social development and genetic factors (e.g., by using twin designs; Crone et al., 2020), and their association with mental health outcomes in a developmental context (Ferschmann et al., 2021).

Crucial in contextualizing individual differences in etiology and maintenance of mental health problems is investigating neurobiological mechanisms of psychopathology from a longitudinal perspective. Specifically, non-linear developmental changes in cortical and subcortical structures may explain why cross-sectional developmental neuroimaging studies may find mixed results depending on the age range of the participants (Wierenga et al., 2014a,b; Mills et al., 2021). With longitudinal designs, we, for example, found that heightened scores on externalizing symptoms were associated with smaller developmental changes in brain structure (Vijayakumar et al., 2014; Oostermeijer et al., 2016; Ambrosino et al., 2017; Bos et al., 2018a; but see Ducharme et al., 2011). Likewise, for internalizing symptoms, such as depression, longitudinal studies revealed associations with aberrant brain development (Whittle et al., 2014; Luby et al., 2016; Bos et al., 2018b). Together, these studies highlight the importance of longitudinal studies for understanding mental health problems and their development.

## Integrating Science and Society

Integrating scientific knowledge about adolescent development into society can be achieved in multiple ways. Popular science books such as “The Adolescent Brain” (Het Puberende Brein, Crone, 2018) and “Inventing ourselves” (Blakemore, 2018) help to reach a wider audience, including policymakers (“science for policy”). In our team, we aim to reach adolescents *via* targeted websites designed for youth^1^ and scientific articles for children (Westhoff et al., 2020a). We also inform youth professionals by designing educational material for elementary and high schools about the developing brain (e.g., http://www.breinkennisleiden.nl/onderwijs), and through our contributions to science-translation reports such as those on differences between boys and girls in learning (report Dutch Educational Council), and UNESCO’s International Science and Evidence-based Education Assessment on the social-emotional learning (Gotlieb et al., 2022).

It is particularly important to include adolescents themselves when forming policies and designing interventions in order to make their participation efforts optimal and to contribute to their sense of autonomy which benefits their mental health (Fuligni, 2019). Especially during mid-adolescence interventions typically tend to fail when they do not align with adolescents’ desired feeling to be respected and accorded status (Yeager et al., 2018). Peer-led interventions to generate positive behavioral changes can be powerful when the complexity of peer relations and social networks are taken into account as well as social learning (e.g., imitation, norms, and positive reinforcement; Veenstra and Laninga-Wijnen, 2022). The next step toward improving these efforts is setting up projects in which adolescents are involved in co-designing and co-creating research (Whitmore and Mills, 2021). Not only does this enrich the context in which scientific findings can be launched and interpreted, but crucially informs researchers in important ways, helping them to improve their research designs and paradigms. In our current projects, adolescent volunteers are also involved in disseminating knowledge to their peers, thereby increasing the likelihood that the information is relevant and interesting for the target audience (e.g., http://www.instagram.com/breinboost). Finally, in several innovative projects we involve societal stakeholders (e.g., teachers, adolescents, policymakers) in the research consortium from the start of the project and create research and knowledge dissemination projects together throughout the project (see e.g., http://www.neurolab.nl/startimpuls, Vandenbroucke et al., 2021).

## Conclusions

In this perspectives article, we provided an overview of ways of characterizing developmental changes during adolescence and their relation to the developing brain. We highlighted the importance of capturing social contextual factors, as social experiences play a crucial role in shaping many developmental trajectories. We further emphasized the importance of longitudinal approaches in developmental studies in identifying predictors of mental health. The social context is increasingly important given that young people today grow up in a highly socially complex environment. The recent COVID-19 pandemic showed how strong the effects of the changing social context are on adolescents’ mental health has been (Orben et al., 2020; Van de Groep et al., 2020; Asscheman et al., 2021; Breaux et al., 2021; Green et al., 2021; Klootwijk et al., 2021). An increased understanding of the effect of social contextual factors on the development and neurobiological mechanisms underlying mental health will inform high stake policy questions, and find their way to daily practice. Throughout this overview, we illustrated the value of collaborative team science to understand adolescent development and the value of integration of science and society to be able to inform policy and practice.

## Author Contributions

All authors co-designed the aims and wrote the article. KZ and IM designed the article aims, outline, figure, and integrated individual author contributions. All authors contributed to the article and approved the submitted version.

## Conflict of Interest

The authors declare that the research was conducted in the absence of any commercial or financial relationships that could be construed as a potential conflict of interest.

## Publisher’s Note

All claims expressed in this article are solely those of the authors and do not necessarily represent those of their affiliated organizations, or those of the publisher, the editors and the reviewers. Any product that may be evaluated in this article, or claim that may be made by its manufacturer, is not guaranteed or endorsed by the publisher.

## Footnotes

^1^ http://www.kijkinjebrein.nl/
